# Hemodialysis patients have signs of a chronic thrombotic burden

**DOI:** 10.1186/s12882-024-03654-3

**Published:** 2024-07-12

**Authors:** Bernd G. Stegmayr, Lennart D. Lundberg

**Affiliations:** Department of Public Health and Clinical Medicine, Umea University, Umea, Sweden

**Keywords:** D-dimer, Interdialytic weight gain, Hemodialysis, Thrombosis, Ultrafiltration

## Abstract

**Background:**

Cardiovascular diseases are the dominant cause of morbidity in hemodialysis (HD) patients. Unless sufficient anticoagulation is used during HD, clotting may appear. The objective was to investigate if levels of fibrin degradation products (D-dimer) were increased before and during HD.

**Methods:**

The combined observational study included 20 patients performing a total of 60 hemodialysis divided into three sessions of low-flux dialysis. None of the patients suffered from any clinically evident thromboembolic event before or during the study. Median bolus anticoagulation (mainly tinzaparin) doses were 84 Units/kg bow. Blood samples were drawn before HD (predialysis), and at 30min and 180min during HD with focus on analyzing D-dimer levels and its relation to interdialytic weight gain (IDWG) and speed of fluid elimination by HD (UF-rate).

**Results:**

Predialysis, D-dimer levels (mean 0.767 ±0.821, min 0.136mg/L) were above the upper reference value in 95% of the sessions. D-dimer levels were lowered at 30min (*p*<0.001) and returned to predialysis levels at 180min. Predialysis D-dimer correlated with NT-pro-BNP, Troponin T, IDWG and UF-rate. Multiple regression analysis revealed that the D-dimer levels were significantly related to IDWG and the UF-rate.

**Conclusions:**

D-dimer levels were elevated in a high proportion predialysis and during HD and related to the IDWG and the UF-rate. Awareness of D-dimer levels and future studies will help clarify if optimization of those variables, besides anticoagulation and biocompatibility measures, will eradicate the repeated subclinical thromboembolic events related to each HD; one reason that may explain organ damage and shortened life span of these patients.

**Supplementary Information:**

The online version contains supplementary material available at 10.1186/s12882-024-03654-3.

## Key learning points

### What was known

Predialysis D-dimers more often are increased in elderly and those with a central dialysis catheter. When blood enters the extracorporeal circuit coagulation appears by contact with the dialyzer membranes and microbubbles of air, forming small clots that pass the air-trap and deposit in organs such as lungs, heart, and brain.

### This study adds

D-dimer levels were increased predialysis and during dialysis in most sessions. This despite a median bolus dose of tinzaparin of 84 Units/kg bow and no symptoms of thromboses. In multivariate analysis D-dimers were related to interdialytic weight gain and to the speed of fluid removed during hemodialysis.

### Potential impact

The chronic tissue damage and increased morbidity may well be counteracted by prescribing follow up of D-dimers that may guide clinician to optimize anticoagulation, limit air exposure, interdialytic weight gain and speed of ultrafiltration.

## Introduction

Compared with the general population chronic hemodialysis (HD) patients have highly increased risks of death caused by bleeding, cardiovascular and thromboembolic events [1–3]. A frequent finding is asymptomatic cerebral small-vessel disease that include silent brain infarction, white matter hyperintensities, and cerebral microbleeds; these conditions are related to the future onset of stroke [4] and cerebral atrophy [5]. Hemodialysis-related acute brain injury, consistent with ischemic injury, was verified by Magnetic Resonance during the HD [6]. Episodes of myocardial ischemia were also detected during HD, [7] which may be related to increased prevalence of myocardial stunning in these patients [8, 9].

In the HD setting, the blood membrane interaction and air contamination activate inflammation, cells, and the coagulation cascade [2, 10–15]. Procoagulant conditions induce clotting [2, 10] and microemboli deposited in various organs of the patient [11] that leads to increased levels of D-dimers being an estimate of thrombus formation in the dialyzer and patient [10, 12–14, 16]. The increased D-dimer levels are independently related to ischemic heart disease [15].

Insufficient anticoagulation may result in partial or total clotting of the extra corporeal circuit (ECC) during the HD [16, 17]. The most common anticoagulants used are intravenous heparin and low molecular weight heparin (LMWH), whereas citrate and heparin coated membranes are other modes [18–20]. Despite the use of recommended doses of anticoagulation for chronic, HD [21] clots may develop in the ECC such as within the dialyzer and venous chamber [17, 18, 20, 22, 23].

Previous studies showed that HD patients more often had increased predialysis levels of D-dimers, [12, 13] more frequent in elderly [12, 13] and those with a central dialysis catheter (CDC) [13].

The objective of the present study was to determine D-dimer levels in chronic HD patients without a history of recent thromboses, and to explore factors that could limit exposure.

## Materials and methods

### Patients

The combined observational study included data from 60 HD sessions performed by 20 (8 women and 12 men) long-term chronic HD patients from the same center (Umea University Hospital, Sweden). All patients were in stable condition without a recent history of infections, embolic events, or active tumors within the latest three months. Their primary renal diagnoses: undefined chronic renal failure (*n* =7), polycystic kidney disease (*n* =3), diabetic nephropathy (*n* =3), glomerulo-nephritis (*n* =3), pyelonephritis (*n* =2), and hypertension (*n* =2). The mean age was 65 years (±10). Women did not differ from men in age (65 ± 12 vs 66 ± 10 years) or vintage time of HD (33 ± 28 vs 43 ± 27 months). The vascular accesses used were central dialysis catheters (CDC, 30 sessions), arteriovenous fistula (AVF, 21 sessions), and AV-graft (AVG, 9 sessions). The mean blood pump flow rate used was 317 ± 45 mLmin^-1^. Additional predialysis data are presented in Tables 1 and 2 and in Supplement Tables 1, 2 and 3. The patients were described in more detail in a previous study that investigated their cardiac condition [24].

**Table 1:** The mean, standard deviation (Std. Dev), median, minimum (min) and maximum (max) values of the predialysis variables months being HD patient (vintage), estimated normal weight (Dry weight), weight gain between dialyses – estimated as interdialytic weight gain (IDWG), low molecular weight heparin dose (LMWH) as Units/patient and Units/kg body weight (bow), duration of HD session, removed fluid by ultrafiltration (UF), and speed of removed fluid in relation to IDWG/hour of HD (UF-ratio)

	N	Mean	Std. Dev		Median	Min-max
Age, years	60	65	10		68	42-80
Vintage, months	60	39	28		44.5	3-108
Dry weight, kg	60	83	22		79.5	49-138
IDWG, in % of bow	60	1.69	1.20		1.76	0-4.69
LMWH, Units/patient^a^	57	6930	2511		6500	3000-12000
LMWH, Units/kg bow^a^	57	84.4	22		84	48.7-150
HD session, hours	60	4.2	0.4		4	3.5-5
UF to be removed, ml	60	1347	898		1500	0-3350
UF-ratio, IDWG/hour	60	0.40	0.29		0.4	0-1.17
Hemoglobin, g/L	60	116	10.4		116.5	96-137

^a^information was missing in 3 series for one patient

**Table 2:** Distribution of D-dimer values given as the mean (±SD) and median (min-max) at predialysis (0min), at 30min, at 180min, and the differences (δ) between two measures. N.S. = not significant

	N	Mean (±SD)	Median (Min. - Max.)	*P*-value
D-dimer _0min_	57	0.77 (0.82)	0.50 (0.14 - 5.54)	Reference
D-dimer _30min_	56	0.66 (0.69)	0.48 (0.01 - 4.57)	<0.001
D-dimer _180min_	57	0.74 (0.73)	0.50 (0.13 - 4.27)	N.S.
δD-dimer _30vs0min_	56	-0.11 (0.38)	-0.03 (-2.19 +0.61)	
δD-dimer _180vs0min_	57	-0.02 (0.23)	-0.03 (-1.27 +0.61)	
δD-dimer _180vs30min_	56	0.09 (0.41)	-0.01 (-0.30 +2.57)	

The local ethics committee in Umea, Sweden, had approved the research protocol (EPN 05-138M, addition date 20071016; EPN2012-42-31M, 20120306). Patients were informed and consented to participate in the study that complied with the Declaration of Helsinki.

Dialyses were done using Fresenius 4008, Fresenius 5008, and Gambro AK200 monitors. LMWH anticoagulation, as bolus dose at start of HD, was given as tinzaparin in 57 HDs and as dalteparin (due to adverse events by tinzaparin) in three HDs.

### Study design

Each patient performed three comparative study dialyses as mid-week sessions. The other dialyses, between the study dialyses, were performed with standard HD or hemodiafiltration (HDF) using various Fresenius dialyzers. All study dialyses were performed with low-flux dialyzers made of polysulfone and of the same size (1.8 sqm), either steam or gamma sterilized and using either a high or low blood level in the venous chamber (air trap).

- Option 1: a dry-stored low-flux dialyzer (F8HPS, Fresenius Medical Care, steam sterilized) with a low blood level in the venous chamber, high enough not to induce alarm (Mode-DL)
- Option 2: a dry stored low-flux F8HPS dialyzer with a high blood level (Mode-DH).
- Option 3: a wet-stored low-flux dialyzer (Rexeed18L, Asahi Kasei Medical, gamma sterilized) with a high blood level (Mode-WH).

All patients were dialyzed by all three modes in a cross-over and randomized order. Low-flux membranes were used to minimize loss of middle molecular or larger size of laboratory markers by clearance through the membrane. Blood samples were drawn before HD and at 30min and 180min of HD. During HD, all samples were collected from the arterial site of the dialysis circuit before entrance into the dialyzer. Blood was collected in citrate and EDTA tubes, centrifuged and plasma stored at -80°C until analysis.

Values during dialysis were adjusted for the change in plasma concentration and erythrocyte volume fraction caused by infusion and/or ultrafiltration in accordance with Schneditz et al [25]. Fluid retention between dialyses was estimated as inter-dialytic weight gain (IDWG) calculated as the percentage increase of body weight between two dialyses [26, 27].

*Laboratory analyses* performed within 2 hours of blood collection were hemoglobin (Hb, ref 100-120 gL^-1^), erythrocyte volume fraction (ref 0.30-0.36), neutrophil granulocytes (Neutr, ref 1.8-6.3 x 10E9L^-1^), lymphocytes (Lymph, ref 1.0-3.5 x 10E9L^-1^), monocytes (Monoc, ref 0.3-1.2 x 10E9L^-1^), eosinophils (Eos, ref 0.07-0.3 x 10E9L^-1^), and thrombocytes particle concentration (TPC, ref 145-348 x 10E9L^-1^). Remaining analyses were performed later (samples stored at -80°C). A specific variable was analyzed at the same time to avoid errors between series. Variables were NT-pro-BNP (ProBNP, ref value <125 ngL^-1^ for patients aged 0-74 and <450 ngL^-1^ for elderly, Roche) and highly sensitive Troponin T (Troponin, ref <15ngL^-1^, Roche). ProBNP above the upper limit of 70,000 ngL^-1^ (of the laboratory) was set as 70,000 ngL^-1^. For these series (those above 70,000), no differences in ProBNP value between predialysis and the 180min data during HD could be calculated.

Other variables were pentraxin 3 (PTX, Perseus Proteomics Inc., Tokyo, Japan, ref <3.5 µg L^-1^), activated complement factor 3 (C3a, ref 20-130 µg L^-1^, according to Ekdahl et al. [28]), thrombin antithrombin complex (TAT, ref <3µg L^-1^, Enzyme Research Laboratories Ltd., Swansea, UK), for C3(H_2_O) i.e., full length complement factor 3 (iC3) with a broken thiol ester, analyzed according to Ekdahl et al. [28] with a tentative reference value of 95-1015 µL^-1^ based on 20 healthy individuals, C-reactive protein (CRP, ref <5 mg L^-1^, Immulite 1000, Siemens Healthcare, Erlangen, Germany), von Willebrand factor (vWF, ref 206-238 U L^-1^, Instrumentation Laboratory), tissue plasminogen activator activity (tPAact, ref 0.2-2 IUmL^-1^, Chromolize tPA activity, Biopool, Umeå, Sweden), tissue plasminogen activator mass (tPAm, ref 1-20 µgL^-1^, Trinity Biotech, Ireland Limited, IDA Business Park, Wicklow, Ireland), plasminogen activator inhibitor 1 mass (PAIm, ref 4-43 µgL^-1^, Tcoag, Trinity Biotech, Ireland Limited, IDA Business Park, Wicklow, Ireland), and fibrin degradation product D-dimer (D-dimer, ref <0.20 mg L^-1^, ACL TOP 700 LAS, 750 LAS, Instrumentation Laboratory).

IDWG was calculated as the percentage of weight gain between two dialyses in relation to the dry body weight of the patient. The removed fluid during HD was defined as ultrafiltration (UF, mL). The UF-rate was defined as the speed of removed fluid given as a rate of the IDWG%/hour of HD, such that if the IDWG was 4% of the body weight a 4-hour dialysis with continuous removal of fluid would result in a removal of 1% of body weight, i.e., 1 IDWG/hour. This would give a UF-rate of 1 using a longitudinal elimination.

### Statistical analysis

Calculations were done for numerical data and differences in concentrations (*delta-*values) present at predialysis (before start of HD), at 30min, and 180min of HD. With a two tailed p value and alfa error of <0.05 and power of 0.80 nineteen pairs are sufficient.

Values were compared as pairs, groups, and aggregated.

Data were analyzed using SPSS (PASW Statistics for Windows, Version 28. Chicago: SPSS Inc.). The Wilcoxon signed rank test was used for paired samples, and Mann-Whitney U test used for independent samples. Correlations were analyzed with the non-parametric Spearman’s test (rho-value). Values are expressed as median (minimal and maximal) and mean (± standard deviation, SD). Significant values were defined as a two-tailed P-value <0.05. The P-value should be put in relation to the extent of secondary analyses, considering the Bonferroni concept of alfa-error for multiple analyses. Since every patient performed all three modes of treatments, the analyses were performed for all aggregate dialyses (*n* =60) and for each specific mode of HD (each *n* =20). Multiple linear regression analyses with D-dimer as dependent factor were performed using the stepwise model, including the variables age and significant variables from the bivariate analysis.

## Results

### Predialysis data

**Fig. 1:**
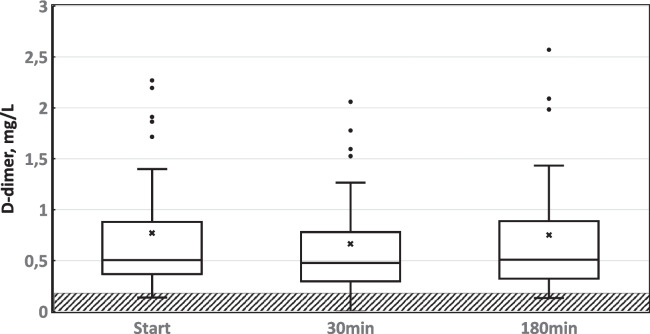
D-dimer median values (CI 95%, and 25% percentile) before HD (Start), at 30min and at 180min of HD. Reference values <0.2 mg/L (hatched area)

D-dimer levels at predialysis were ≥ 0.20 mg/l in 95% of sessions and ≥ 0.50 mg/L in 53%. Other variables with more than 50% samples above the upper reference value at predialysis were ProBNP (100%), iC3 (98%), troponin (96%), PTX (78%), PAIm (63%), TAT (58%) and vWF (56%). D-dimer values did not differ between men and women at predialysis (0min), at 30min or at 180min of dialysis. As a result, sex was not entered in the multiple regression analysis. Further data are given in Table 2, Fig. 1, and Supplement Table 1-3.

*Bivariate correlation analyses* between variables are shown in Supplement Table 3. Predialysis D-dimer correlated with age (*rho*=0.309, *p*=0.019), IDWG (*rho*=0.278, *p*=0.036), the UF-rate (*rho*=0.332, *p*=0.012), proBNP_0min_ (*rho*=0.286, *p*=0.034), Troponin_0min_ (*rho*=0.385, *p*=0.004), tPAact_0min_ (*rho*=0.347, *p*=0.008), and TAT_0min_ (*rho*=0.494, *p*<0.001), and inversely with the PAIm_0min_ (*rho*=-0.273, *p*=0.04).

### Impact of HD (Supplement Tables 1-3, Fig. 1)

When comparing laboratory variables between the three different modes of dialyses, the variables did not differ except for a significant reduction of lymphocytes and eosinophils at 30min with Mode-DL compared to both Mode-DH and Mode-WH.

For aggregated data, the D-dimer level at 30min was lowered from predialysis (0min) (p<0.001), whereas it increased back to the predialysis level at 180min of HD (*p*=0.111, Fig 1).

Supplement Table 1 shows the change from the predialysis, and at 30 and 180 min of HD for thrombocytes and for various types of leukocytes. Supplement Table 2 shows variations in additional variables for inflammation, coagulation, and fibrinolysis.

Supplement Table 2 shows statistical comparisons for changes from predialysis (0min) in coagulation and fibrinolytic variables during the HD.

*Bivariate comparison* of D-dimers (Supplement Table 3) at 30min of HD as the dependent factor correlated with UF-rate (*rho*=0.331, *p*=0.013), IDWG (*rho*=0.304, *p*=0.023), vintage time ( *rho*=0.343, *p*=0.010), tPAact_30min_ (*rho*=0.386, *p*=0.003), tPAm_30min_ (*rho*=0.386, *p*=0.003), TAT_30min_ (*rho*=0.444, *p*<0.001), and the change in eosinophils _Diff 30 vs 0min_ (*rho*=0.326, *p*=0.018).

Bivariate comparison of D-dimers at 180min of HD as the dependent factor (Supplement Table 3) correlated with the UF-rate (*rho*=0.277, *p*=0.037), ProBNP_180min_ (*rho*=0.280, *p*=0.040), Troponin_180min_ (*rho*=0.320, *p*=0.018), TAT_180min_ (*rho*=0.424, *p*=0.001), and inversely with the change in Lymph _Diff 30 vs 0min_ (*rho*=-0.314, *p*=0.024), and Monoc _Diff 30 vs 0min_ (*rho*=-0.345, *p*=0.012).

### Other variables

After HD, the dialyzers were visually graded for clotting. ‘No signs of clotting’ were noted in 39 dialyses, ‘a few stripes’ in 5, and ‘moderate stripes’ in 11 dialyses (grading was missing in 5). No clinical evident thromboembolic event was detected during the study.

Access comparisons in relation to D-dimer showed that cases with arteriovenous fistula (AVF) had similar levels as those with central dialysis catheter (CDC) but lower values than arteriovenous graft (AVG) cases at predialysis (median 431 vs 769, *p*=0.043) and at 180min (median 390 vs 795, *p*=0.01).

iC3 was increased above the upper reference in 98% of dialyses at 30min and 100% at 180min.

### Multiple regression analysis

Except for age, variables included in the multiple repression analyses were those significant in the bivariate analyses.

Stepwise mode with D-dimer levels at predialysis (0min) as the dependent factor showed a significant relation (*r*=0.664, r-square 0.441, *p*=0.013) including the variables tPAact_0min_ (*p*<0.001), TAT_0min_ (*p*=0.001), UF-rate (*p*=0.004) and the IDWG (*p*=0.013).

The D-dimer levels at 30min as the dependent variable showed a significant relation (*r*=0.640, r-square 0.410, *p*=0.002) with the variables tPAact_30min_ (*p*<0.001), the UF-rate (*p*<0.001) and the tPAm_30min_ (*p*=0.014).

When D-dimer at 180min was the dependent variable and related to predialysis variables (as an estimate of initial conditions), the analysis revealed a significant relation (*r*=0.712, r-square 0.508, *p*=0.001) with the variables tPAact_0min_ (*p*<0.001), TAT_0min_ (*p*<0.001), UF-rate (p<0.001), and IDWG (*p*=0.001).

When D-dimer at 180min was the dependent variable and related to the variables appearing at 30min and 180min HD, the analysis revealed the significant model (*r*=0.674, r-square 0.454, *p*=0.038) that included the change in lymphocytes _Diff 30 vs 0min_ (*p*=0.009) and monocytes _Diff 30 vs 0min_ (*p*=0.007), Troponin_180min_ (*p*=0.023), and TAT_180min_ (*p*=0.038).

## Discussion

We had hypothesized that chronic HD patients without a history of recent thromboses may suffer from significant thromboembolic events measured by D-dimer levels. The aim was also to explore factors that could limit such exposure.

### Key results

The present study showed that chronic HD patients without a recent history of thromboembolic disease had increased D-dimer values at predialysis before HD, in almost all sessions. The reference level of <0.20 mg/L set by the manufacturer but also by the local hospital, may seem low. However, based on a local population study, [29] controls had a mean of 0.20 mg/L (IQR 0.14-0.30) while in a surgical study, preoperative patients for cholecystectomy had a mean of 0.02 mg/L [30]. A lower reference level will minimize the risk of missing follow-up of a recent thrombosis [31]. Therefore, our generalized interpretation is that the increased D-dimer levels express residual thrombo-emboli still two days after the previous HD that may develop into fibrotic tissue and cumulative organ damage. Significant variables related to D-dimer in our study, that can be implemented, are IDWG and the UF-rate.

The significant reduction in D-dimers at 30 minutes of HD is most probably due to adherence of D-dimers and other fibrin degradation products within the dialyzer. This is indicated by the relation between the graded estimation of clotting of dialyzers after HD and the more pronounced percentage reduction of D-dimer at 30min of HD. Again, UF-rate seems to be of importance for the outcome.

The 180min D-dimer level that increased back to the predialysis level at 180min is interpreted to reflect new deposits of clots in the bloodline and body induced by reactivated fibrinolysis. This appeared despite the prescription of anticoagulation in doses being more than double of the recommended dosage of approximately 25 Units LMWH/kg body weight [21]. Elevated D-dimer and clotted fibers of dialyzers may help guide the anticoagulation doses.

Although the bivariate analysis indicated that predialysis D-dimer was related to age and myocardial strain (proBNP and Troponin), those variables were lost in the multivariate analysis. Instead, IDWG and UF-rate were significant factors; these variables may be improved by limited fluid intake and prolonged HD time/session. The relation of predialysis D-dimer to tPA and TAT levels is interpreted to reflect the activity of fibrinolysis.

The 180min D-dimer level seems to depend on the predialysis variables D-dimer (as retained clots) and activated fibrinolysis as shown by tPA and TAT. These variables may be favorably influenced by optimized anticoagulation. Another significant factor was the UF-rate. This is in line with others that reported an increase in D-dimer post-dialyzer when studying online post-dilutional hemodiafiltration [32]. Our interpretation is that more extensive ultrafiltration performed by a shorter dialysis time increases hematocrit, shear stress, and platelet activation, and thereby induces more clotting. The interaction of HD on reduction of lymphocytes at 30min may be improved by biocompatibility measures, since the innate system responds rapidly to infection and injury, involving lymphocytes [33]. In a previous study we found that the exposure of microbubbles of air was related to a greater lymphocyte reduction [24]. In the present study, the Mode-DL had a more extensive lymphocyte reduction than the other Modes. In another study, the Mode-DL showed a significantly greater exposure of air microbubbles in the return bloodline of the patients than did the other Modes [34]. Since oxidative stress activates lymphocytes and procoagulants, [35–37] this together may explain the interference of air bubble containing microemboli in the body such as lungs, heart and the brain [11]. During HD, blood is exposed to air contamination throughout most parts of the extracorporeal circuit, [38–40] which activates the blood membrane interaction and the coagulation system [2, 41]. The importance of oxygen contamination of the bloodline is further strengthened by the high iC3 levels in the present study; iC3 is a breakdown product of C3 upon exposure of blood to oxygen [28]. Our iC3 levels were above the upper normal at almost all measurements.

### Comparison with previous studies

As shown by others, [12, 13, 42] and even more prevalent, we found overall increased predialysis levels of D-dimer in the HD patients devoid of symptoms of thromboses. In contrast to others, [13, 42] we showed further increased levels of D-dimer for those with AV-graft and not those with CDC or AVF. The increase of D-dimer in the AVG group may be related to a general vascular problem in these patients. However, the data do not rule out that thromboses also may derive from the site of AVG and CDC.

### Study limitations

We did not expect such an extensive prevalence of increased D-dimer levels at predialysis. Therefore, we did not plan for a follow-up measure of D-dimers also between dialyses and before the next standard dialysis. A limiting consequence was that the predialysis values in the present study were depending on the different standard dialyses that the patients performed between the study dialyses.

### Clinical implications

Based on the present data, we recommend awareness of D-dimer levels and preventive measures to limit clotting by further improving biocompatibility of the extracorporeal circuit such as dialyzer membrane properties, [10, 43, 44] minimizing of air contamination of the bloodlines, [11, 45, 46] and optimizing anticoagulation routines during and between hemodialysis [10, 20, 43, 47]. Direct measures could be to motivate patients to limit the IDWG [24, 26] and to prescribe a lower UF-rate/hour. All measures that may counteract the repeated subclinical thromboembolic events related to each HD and instead counteract organ damage and help extend life span of these patients.

In conclusion, D-dimer levels were elevated in most predialysis and hemodialysis sessions and related to IDWG and UF-rate.

### Supplementary Information

Supplementary Material 1. 

Supplementary Material 2.

Supplementary Material 3. 

## Data Availability

The data underlying this research will be made available upon reasonable request to the corresponding author.
