# Supplementary material for: Hemodialysis patients have signs of a chronic thrombotic burden
Source: BMC Nephrol. 2024 Jul 12;25:223. doi: 10.1186/s12882-024-03654-3 (PMC11245813; doi:10.1186/s12882-024-03654-3)
Supplement: Supplementary file 1 — Supplementary Material 1. [file 12882_2024_3654_MOESM1_ESM.docx]

Supplement Table 1: Predialysis values and changes in thrombocyte particle concentration (TPC), neutrophils (Neutr), lymphocytes (Lymph), eosinophils (Eos) and monocytes (Monoc) from the start (0min) with a focus on levels at 30min, as well as at 180 min for TPC. Also calculated were the change (Diff) at 30min from 0min (30-0) and at 180min from 0min (180-0), respectively. Wilcoxon paired statistics (p).

|  | **N** | | **Mean** | **Median** | **Std. Deviation** | **Minimum** | **Maximum** | **p=** |
| --- | --- | --- | --- | --- | --- | --- | --- | --- |
|  | Valid | Missing |  |  |  |  |  |  |
| TPC_0min_ | 60 | 0 | 234 | 210 | 102 | 52 | 502 | versus |
| TPC_30min_ | 59 | 1 | 224 | 207 | 96 | 51 | 462 | <0.001 |
| TPC_180min_ | 60 | 0 | 225 | 201.5 | 93 | 55 | 458 | <0.001 |
| Neutr_0min_ | 56 | 4 | 4.22 | 3.85 | 1.81 | 1.8 | 9.7 | versus |
| Neutr_30min_ | 56 | 4 | 4.15 | 3.95 | 1.70 | 1.68 | 8.9 | 0.038 |
| Lymph_0min_ | 56 | 4 | 1.33 | 1.2 | 0.54 | 0.7 | 3.4 | versus |
| Lymph_30min_ | 56 | 4 | 1.22 | 1.10 | 0.57 | 0.4 | 3.2 | <0.001 |
| Eos_0min_ | 56 | 4 | 0.29 | 0.2 | 0.20 | 0.03 | 1 | versus |
| Eos_30min_ | 56 | 4 | 0.29 | 0.20 | 0.23 | 0.06 | 1.1 | 0.003 |
| Monoc_0min_ | 56 | 4 | 0.58 | 0.55 | 0.21 | 0.2 | 1.2 | versus |
| Monoc_30min_ | 56 | 4 | 0.46 | 0.49 | 0.17 | 0.2 | 0.8 | <0.001 |
| TPC_Diff30-0min_ | 59 | 1 | -11.4 | -7 | 17.2 | -66 | 12 | <0.001 |
| TPC_Diff180-0min_ | 60 | 0 | -9.57 | -6.50 | 18.9 | -84 | 21 | <0.001 |
| Neutr_Diff30-0min_ | 55 | 5 | -0.15 | -0.10 | 0.63 | -1.85 | 1.76 | 0.038 |
| Lymph_Diff30-0min_ | 55 | 5 | -0.14 | -0.1 | 0.24 | -1.08 | 0.22 | <0.001 |
| Eos_Diff30-0min_ | 55 | 5 | -0.004 | -0.005 | 0.149 | -0.2 | 1.02 | 0.003 |
| Monoc_Diff30-0min_ | 55 | 5 | -0.13 | -0.10 | 0.17 | -0.6 | 0.2 | <0.001 |
