# Supplementary material for: Hemodialysis patients have signs of a chronic thrombotic burden
Source: BMC Nephrol. 2024 Jul 12;25:223. doi: 10.1186/s12882-024-03654-3 (PMC11245813; doi:10.1186/s12882-024-03654-3)
Supplement: Supplementary file 2 — Supplementary Material 2. [file 12882_2024_3654_MOESM2_ESM.docx]

Supplement Table 2: Predialysis (0min) and follow-up values at 30min and 180min for the following variables: von Willebrand Factor (vWF), complement factor 3 activity (C3a), pentraxin (PTX), C-reactive protein (CRP), plasminogen activator inhibitor 1 mass (PAImass), tissue plasminogen activator (tPAact), tissue plasminogen activator mass (tPAmass), thrombin antithrombin complex (TAT), and complement factor 3 (iC3).

The p-value is based on comparison with predialysis value noted as ‘versus’. Wilcoxon paired statistics (p). NS= Not significant

|  | N valid | Mean | Std. Deviation | Median | Min-Max | P for value vs 0min |
| --- | --- | --- | --- | --- | --- | --- |
| Hemoglobin_0min_ | 60 | 116 | 10.4 | 116.5 | 96-137 |  |
| vWF_0min_ | 57 | 289 | 157 | 266 | 81-739 | versus |
| vWF_30min_ | 54 | 294 | 147 | 271 | 86-683 | NS |
| vWF_180min_ | 57 | 316 | 165 | 308 | 102-809 | 0.037 |
| C3a_0min_ | 60 | 164 | 687 | 70 | 31-5392 | versus |
| C3a_30min_ | 59 | 188 | 565 | 105 | 41-4443 | <0.001 |
| C3a_180min_ | 60 | 91 | 33 | 83 | 41-215 | 0.001 |
| PTX_0min_ | 60 | 6.35 | 4.3 | 5.5 | 2.2-25 | versus |
| PTX_30min_ | 59 | 7.8 | 5.1 | 6.7 | 2.7-30 | <0.001 |
| PTX_180min_ | 60 | 9.9 | 5.6 | 8.5 | 3.4-34 | <0.001 |
| CRP_0min_ | 57 | 5.2 | 9.8 | 2.3 | 0.3-68 | versus |
| CRP_30min_ | 56 | 5.1 | 10 | 2 | 0-71 | NS |
| CRP_180min_ | 57 | 5.1 | 11 | 1.9 | 0.3-77 | NS |
| PAImass_0min_ | 57 | 57 | 31 | 64 | 10-120 | versus |
| PAImass_30min_ | 56 | 50 | 29 | 48 | 9-113 | <0.001 |
| PAImass_180min_ | 57 | 54 | 31 | 53 | 12-117 | 0.018 |
| tPAact_0min_ | 57 | 0.70 | 0.44 | 0.62 | 0.07-2.91 | versus |
| tPAact_30min_ | 56 | 1.32 | 0.88 | 1.17 | 0-3.36 | <0.001 |
| tPAact_180min_ | 57 | 0.92 | 0.47 | 0.82 | 0-2.51 | <0.001 |
| tPAmass_0min_ | 57 | 8.4 | 6.0 | 7.2 | 2.1-38 | versus |
| tPAmass_30min_ | 56 | 10.2 | 6.7 | 8.8 | 0.0-34 | <0.001 |
| tPAmass_180min_ | 57 | 10.4 | 6.1 | 8.2 | 3.0-30 | <0.001 |
| TAT_0min_ | 60 | 4.51 | 6.22 | 3.63 | 0.10-49 | versus |
| TAT_30min_ | 59 | 2.49 | 1.24 | 2.62 | 0.10-6.7 | <0.001 |
| TAT_180min_ | 60 | 2.62 | 1.37 | 2.42 | 0.09-7.4 | <0.001 |
| iC3 _0min_ | 60 | 8078 | 9118 | 5908 | 610-65380 | versus |
| iC3 _30min_ | 60 | 12064 | 30584 | 5476 | 962-236891 | NS |
| iC3_180min_ | 60 | 6946 | 4426 | 5479 | 1091-27688 | NS |
