# Supplementary material for: Hemodialysis patients have signs of a chronic thrombotic burden
Source: BMC Nephrol. 2024 Jul 12;25:223. doi: 10.1186/s12882-024-03654-3 (PMC11245813; doi:10.1186/s12882-024-03654-3)
Supplement: Supplementary file 3 — Supplementary Material 3. [file 12882_2024_3654_MOESM3_ESM.docx]

Supplement Table 3: Spearman’s correlation between D-dimer samples before (0min), at 30min and at 180min of HD versus various variables for the same time points. Also included are D-dimer relations to the difference (Diff) for Neutr, Lymph and Eos between 30min and 0min. UF-ratio is calculated as removed extent of IDWG/hour of dialysis.

|  | **D-dimer 0min** | | | **D-dimer at 30min** | | | **D-dimer at 180min** | | |
| --- | --- | --- | --- | --- | --- | --- | --- | --- | --- |
|  | Correlation Coefficient | Sig. (2-tailed) | N | Correlation Coefficient | Sig. (2-tailed) | N | Correlation Coefficient | Sig. (2-tailed) | N |
| LMWH,  Units/kg Bow | -0.049 | 0.724 | 54 | 0.056 | 0.692 | 53 | -0.068 | 0.626 | 54 |
| LMWH,  Units/dialysis | -0.311 | 0.022 | 54 | -0.206 | 0.139 | 53 | -0.364 | 0.007 | 54 |
| IDWG, %/Bow | 0.278 | 0.036 | 57 | 0.304 | 0.023 | 56 | 0.210 | 0.118 | 57 |
| Age, years | 0.309 | 0.019 | 57 | 0.284 | 0.034 | 56 | 0.326 | 0.013 | 57 |
| HD session, hours | -0.199 | 0.139 | 57 | -0.108 | 0.427 | 56 | -0.272 | 0.041 | 57 |
| UF-ratio | 0.332 | 0.012 | 57 | 0.331 | 0.013 | 56 | 0.277 | 0.037 | 57 |
| Vintage. months | 0.245 | 0.066 | 57 | 0.343 | 0.010 | 56 | 0.213 | 0.111 | 57 |
| **Predialysis (0min) Lab-values:** | | | | | | | | | |
| ProBNP_0min_ | 0.286 | 0.034 | 55 | 0.305 | 0.023 | 55 | 0.290 | 0.034 | 55 |
| Troponin_0min_ | 0.385 | 0.004 | 55 | 0.368 | 0.006 | 55 | 0.351 | 0.009 | 55 |
| PTX_0min_ | 0.178 | 0.185 | 57 | 0.270 | 0.044 | 56 | 0.257 | 0.054 | 57 |
| PAIm_0min_ | -0.273 | 0.04 | 57 | -0.223 | 0.098 | 56 | -0.248 | 0.063 | 57 |
| tPAact_0min_ | 0.347 | 0.008 | 57 | 0.224 | 0.096 | 56 | 0.305 | 0.021 | 57 |
| tPAm_0min_ | 0.235 | 0.078 | 57 | 0.174 | 0.201 | 56 | 0.194 | 0.147 | 57 |
| vWF_0min_ | 0.125 | 0.352 | 57 | 0.142 | 0.295 | 56 | 0.08 | 0.553 | 57 |
| CRP_0min_ | 0.152 | 0.259 | 57 | 0.179 | 0.188 | 56 | 0.16 | 0.233 | 57 |
| D-dimer_0min_ | 1 | . | 57 | 0.771 | <0.001 | 56 | 0.934 | <0.001 | 57 |
| TAT_0min_ | 0.494 | <0.001 | 57 | 0.601 | <0.001 | 56 | 0.571 | <0.001 | 57 |
| C3a_0min_ | 0.237 | 0.076 | 57 | 0.176 | 0.193 | 56 | 0.194 | 0.147 | 57 |
| TPC_0min_ | 0.096 | 0.476 | 57 | -0.003 | 0.982 | 56 | 0.116 | 0.389 | 57 |
| Hb_0min_ | 0.122 | 0.365 | 57 | 0.049 | 0.720 | 56 | 0.086 | 0.524 | 57 |
| **30min dialysis Lab-values:** | | | | | | | | | |
| PAIm_30min_ | -0.19 | 0.161 | 56 | -0.157 | 0.246 | 56 | -0.19 | 0.161 | 56 |
| tPAact_30min_ | 0.265 | 0.048 | 56 | 0.386 | 0.003 | 56 | 0.255 | 0.058 | 56 |
| tPAm_30min_ | 0.235 | 0.081 | 56 | 0.366 | 0.006 | 56 | 0.204 | 0.132 | 56 |
| vWF_30min_ | 0.022 | 0.872 | 54 | -0.036 | 0.797 | 54 | -0.014 | 0.922 | 54 |
| CRP_30min_ | 0.056 | 0.68 | 56 | 0.263 | 0.050 | 56 | 0.052 | 0.701 | 56 |
| D-dimer_30min_ | 0.771 | <0.001 | 56 | 1 | . | 56 | 0.788 | <0.001 | 56 |
| TAT_30min_ | 0.345 | 0.009 | 56 | 0.444 | <0.001 | 56 | 0.430 | <0.001 | 56 |
| PTX_30min_ | 0.134 | 0.324 | 56 | 0.221 | 0.102 | 56 | 0.218 | 0.107 | 56 |
| C3a_30min_ | 0.265 | 0.049 | 56 | 0.169 | 0.214 | 56 | 0.252 | 0.062 | 56 |
| TPC_30min_ | -0.007 | 0.959 | 56 | 0.095 | 0.485 | 56 | 0.026 | 0.849 | 56 |
| Hb_30min_ | 0.016 | 0.908 | 57 | -0.056 | 0.683 | 56 | -0.018 | 0.892 | 57 |
| Neutr _Diff 30 vs 0min_ | -0.157 | 0.265 | 52 | -0.003 | 0.981 | 52 | -0.178 | 0.207 | 52 |
| Lymph _Diff 30 vs 0min_ | -0.274 | 0.049 | 52 | -0.208 | 0.138 | 52 | -0.314 | 0.024 | 52 |
| Eos _Diff 30 vs 0min_ | 0.029 | 0.84 | 52 | 0.326 | 0.018 | 52 | 0.102 | 0.471 | 52 |
| Monoc _Diff 30 vs 0min_ | -0.345 | 0.012 | 52 | -0.145 | 0.305 | 52 | -0.345 | 0.012 | 52 |
| **180min dialysis Lab-values:** | | | | | | | | | |
| ProBNP_180min_ | 0.292 | 0.032 | 54 | 0.304 | 0.025 | 54 | 0.280 | 0.040 | 54 |
| Troponin_180min_ | 0.381 | 0.004 | 54 | 0.358 | 0.008 | 54 | 0.320 | 0.018 | 54 |
| PTX_180min_ | 0.078 | 0.563 | 57 | 0.162 | 0.234 | 56 | 0.145 | 0.282 | 57 |
| PAIm_180min_ | -0.215 | 0.108 | 57 | -0.170 | 0.210 | 56 | -0.175 | 0.193 | 57 |
| tPAact_180min_ | 0.181 | 0.178 | 57 | 0.197 | 0.146 | 56 | 0.167 | 0.216 | 57 |
| tPAm_180min_ | 0.263 | 0.048 | 57 | 0.207 | 0.126 | 56 | 0.219 | 0.102 | 57 |
| vWF_180min_ | 0.009 | 0.948 | 57 | -0.032 | 0.816 | 56 | -0.027 | 0.844 | 57 |
| CRP_180min_ | 0.11 | 0.415 | 57 | 0.131 | 0.336 | 56 | 0.116 | 0.391 | 57 |
| D-dimer_180min_ | 0.934 | <0.001 | 57 | 0.788 | <0.001 | 56 | 1 | . | 57 |
| TAT_180min_ | 0.287 | 0.03 | 57 | 0.178 | 0.189 | 56 | 0.424 | 0.001 | 57 |
| C3a_180min_ | 0.168 | 0.212 | 57 | 0.119 | 0.382 | 56 | 0.186 | 0.167 | 57 |
| TPC_180min_ | -0.002 | 0.987 | 57 | 0.066 | 0.631 | 56 | -0.057 | 0.675 | 57 |
| Hb_180min_ | 0.055 | 0.686 | 57 | -0.014 | 0.916 | 56 | 0 | 0.998 | 57 |
